# Identifying hybridization and admixture using SNPs: application of the DArTseq platform in phylogeographic research on vertebrates

**DOI:** 10.1098/rsos.161061

**Published:** 2017-07-19

**Authors:** Jane Melville, Margaret L. Haines, Katja Boysen, Luke Hodkinson, Andrzej Kilian, Katie L. Smith Date, Dominique A. Potvin, Kirsten M. Parris

**Affiliations:** 1 Department of Sciences, Museum Victoria, Carlton, Victoria 3052, Australia; 2 Diversity Arrays Technology, University of Canberra, Bruce, Australian Capital Territory 2617, Australia; 3 School of Ecosystem and Forest Sciences, The University of Melbourne, Parkville, Victoria 3010, Australia

**Keywords:** DArTseq, genomics, hybridization, population genetics, phylogeography, SNPs

## Abstract

Next-generation sequencing (NGS) approaches are increasingly being used to generate multi-locus data for phylogeographic and evolutionary genetics research. We detail the applicability of a restriction enzyme-mediated genome complexity reduction approach with subsequent NGS (DArTseq) in vertebrate study systems at different evolutionary and geographical scales. We present two case studies using SNP data from the DArTseq molecular marker platform. First, we used DArTseq in a large phylogeographic study of the agamid lizard *Ctenophorus caudicinctus*, including 91 individuals and spanning the geographical range of this species across arid Australia. A low-density DArTseq assay resulted in 28 960 SNPs, with low density referring to a comparably reduced set of identified and sequenced markers as a cost-effective approach. Second, we applied this approach to an evolutionary genetics study of a classic frog hybrid zone (*Litoria ewingii–Litoria paraewingi*) across 93 individuals, which resulted in 48 117 and 67 060 SNPs for a low- and high-density assay, respectively. We provide a docker-based workflow to facilitate data preparation and analysis, then analyse SNP data using multiple methods including Bayesian model-based clustering and conditional likelihood approaches. Based on comparison of results from the DArTseq platform and traditional molecular approaches, we conclude that DArTseq can be used successfully in vertebrates and will be of particular interest to researchers working at the interface between population genetics and phylogenetics, exploring species boundaries, gene exchange and hybridization.

## 1. Introduction

Population genetics and phylogeographic research have shown that many species consist of multiple, highly divergent genetic lineages, with evidence of hybridization and introgression between these lineages [1]. As such, genetic analyses have become a cornerstone of species delimitation and evolutionary biology [2]. However, both theoretical and empirical work show that traditional genetic approaches may have a number of biases and shortfalls related to the stochasticity of evolutionary processes operating at the population scale, and that increased genetic sampling across the genome is fundamental for improved accuracy [3].

Researchers of phylogeography and evolutionary genetics have turned to next-generation sequencing (NGS) as a means to generate multi-locus data for non-model organisms in a time-efficient and cost-effective process [4,5]. Advances in NGS have led to the development of large-scale sequencing arrays based on reduced genome representations, which may provide thousands of markers densely covering the genome. Examples include restriction site-associated DNA sequencing (RADseq), genotyping by sequencing (GBS) and others [6,7]. Many of these NGS methods depend on restriction enzymes to produce a reduced representation of a genome, one such method is DArTseq (Diversity Array Technology sequencing).

DArT was first developed in early 2000 [8] and allowed for the detection of DNA polymorphisms without the need for prior DNA sequence information. The technology was based on hybridization and solid-state surfaces, rather than relying on resolving DNA polymorphisms through electrophoretic gel separation, and thus helped to improve both throughput and accuracy. Today, DArT can be used in combination with NGS, together referred to as DArTseq [9]. In brief, genome reduction is achieved by a combination of endonucleases that specifically target low-copy DNA areas, rather than repetitive DNA fragments [10]. This allows for detection of a high number of informative SNPs across the genome. The result is a genomic ‘representation’, comprising both constant and polymorphic fragments across individuals. NGS of these ‘representations’ reveals the sequence (approx. 70 bp) of an informative DNA fragment and each individual's state compared with all others, namely (i) homozygosity with reference allele, (ii) homozygosity with alternate allele, or (iii) heterozygosity, comprising both a reference and an alternate SNP allele.

DArTseq has been applied across numerous plant species, due to high throughput capabilities, genome coverage and interspecific transferability; however, to date it has rarely, albeit successfully, been used in animal systems [11–15]. Here, we present results of two case studies using SNPs from the DArTseq molecular marker platform. We selected two systems that have been well studied with the application of traditional molecular techniques, but that differ in both geographical scope and evolutionary timescales. The first case study of the agamid lizard *Ctenophorus caudicinctus* has a phylogeographic focus. *Ctenophorus caudicinctus* is broadly distributed across arid Australia, and a multi-gene study previously identified deep phylogeographic structure with evidence of introgression and hybridization between lineages [16]. In our second case study, we investigate a well-studied frog hybrid zone between *Litoria ewingii* and *Litoria paraewingi* in southeastern Australia, which has been of particular interest for our understanding of how barriers to gene flow are maintained [17]. We detail the applicability of a genome complexity reduction approach using restriction enzymes (DArTseq) in these case studies using a variety of analytical approaches, including PCoAs and Bayesian model-based clustering. We also use a conditional likelihood approach, to provide a new method to investigating phylogenetic relationships with DArTseq data. We then compare results with those from traditional sequencing methods.

## 2. Material and methods

### 2.1. Study systems

#### 2.1.1. Case study 1

**Figure 1.**
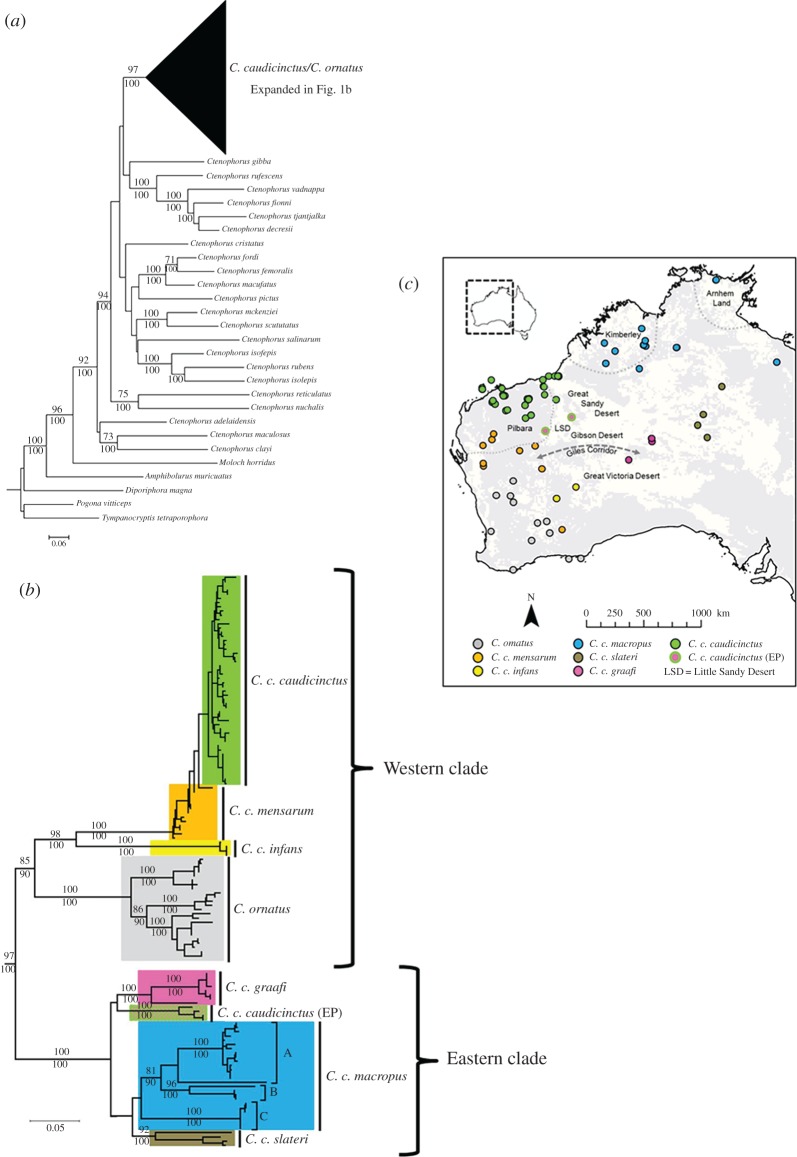
Previously published maximum-likelihood phylogenetic tree for *C. ornatus* and the six subspecies of *C. caudicinctus* based on approximately 1400 bp mtDNA [16]. ML bootstraps greater than 70% (above) and Bayesian posterior probabilities greater than 90% (below) are provided on branches. Colours designate clades, which are mapped.

*Ctenophorus caudicinctus* is a rock-dwelling agamid lizard that is widely distributed in arid and semi-arid regions across the western half of Australia (figure 1). It occupies desert ranges and rocky outcrops, with large expanses of sand deserts believed to provide barriers to dispersal [16]. A recent phylogeographic study, incorporating mtDNA and five nuclear genes, found two deeply divergent mtDNA clades within *C. caudicinctus*—an eastern and western clade—separated by the Western Australian sand deserts [16]. Phylogenetic analyses of the nuclear DNA datasets generally support major mtDNA clades; however, resolution was poor across nuclear loci, probably due to incomplete lineage sorting. Divergences were estimated to have occurred during the Miocene followed by secondary contact during the Pliocene, with evidence of introgression and hybridization between clades.

#### 2.1.2. Case study 2

**Figure 2.**
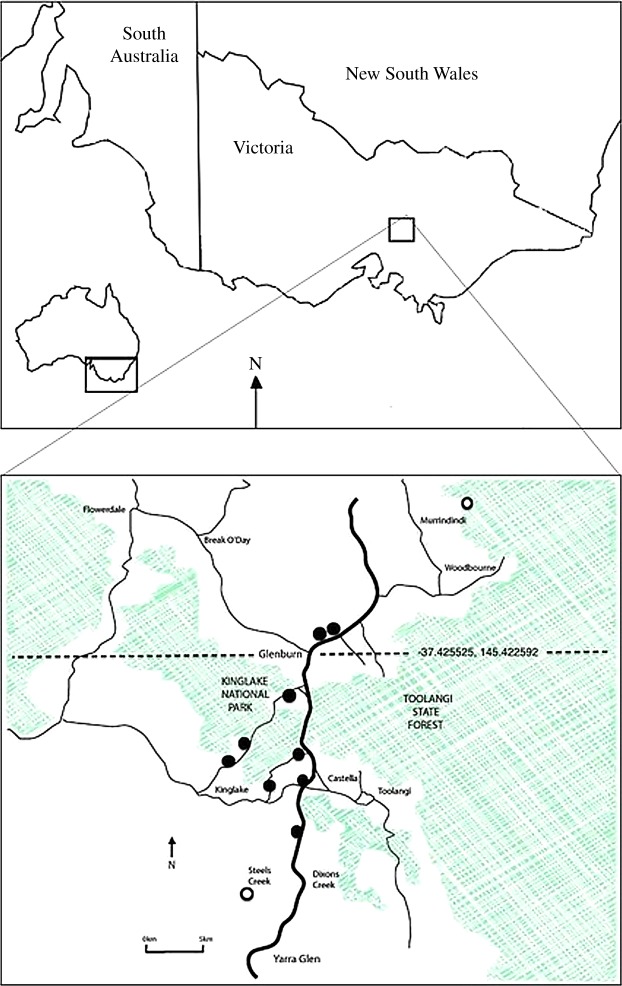
Map of the previously studied hybrid zone between the frogs *L. ewingii* and *L. paraewingi* in Victoria, southeastern Australia. Bold black line indicates the ‘Glenburn transect’, including the locations of 11 sites sampled 2007–2013 [17]. Shaded areas indicate forested regions versus cleared land (unshaded).

*Litoria ewingii–L. paraewingi* is a classic frog hybrid zone in southeastern Australia (figure 2), which has been studied over the last 50 years [17]. This hybrid zone is considered one of the most comprehensively studied amphibian hybrid zones, containing a significant amount of historically collected data. A recent study incorporating mtDNA and eight nuclear microsatellite markers found that these species are genetically distinct and the level of hybridization within the contact zone is low, with the majority of admixed individuals representing later generation hybrids [17]. This research concluded that the *L. ewingii–L. paraewingi* hybrid zone is best characterized as a tension zone, due to the narrow cline width, concordant genetic clines and low levels of hybridization.

### 2.2. Sample preparation

We used genomic DNA extractions from previous studies (*C. caudicinctus* [16]; *Litoria* spp. [17]). Samples to be included in this study were selected to reflect the geographical and genetic diversity uncovered in these previous research projects. Ninety-four samples were selected for each case study, resulting in preparation of one 96-well plate each for subsequent DArTseq assays (the remaining two wells are used for standards in the assays). All *C. caudicinctus* DNA samples were extracted from liver samples, while two *Litoria* spp. DNA samples were extracted from liver and 92 from toe-clips. In addition to the target species, we also included seven samples of *Ctenophorus ornatus* in the *C. caudicinctus* plate, because the previous molecular work based on mtDNA had suggested that these species are paraphyletic. Additionally, we included two *Litoria verreauxii* samples in the *Litoria* plate, because this species has been shown to hybridize with *L. ewingii*, and we wanted to be able to remove any hybrids involving *L. verreauxii* from further analyses, as was done by Smith *et al.* [17]. We also included five samples of *L. ewingii* and four samples of *L. paraewingi* from outside the hybrid zone to provide us with the genetic profile of each parental species.

It is recommended that samples used for DArTseq assays are free of contamination either with RNA or with DNA from other species [7]. This has been found to be a particular problem in aquatic and marine organisms [18,19]. In our second case study, toe-clips from the frogs posed such a problem. In such study systems, cleaning surfaces prior to sample collection may not wholly address this problem. One possibility might be downstream protocols to screen out microbial genetic contamination. For example, NCBI viral and bacterial sequence databases can be used in BLAST searches to detect contamination and all matching loci excluded from the final dataset [18]. In our case, the BLAST search of marker sequences against bacterial and viral databases has yielded no hits. This result can be attributed to DArTsoft14 DArT PL's proprietary software's capacity to perform this ‘filtering’ of viral and/or bacterial sequences in SNP marker selection. It has been achieved through training the program to distinguish allelic sequence variants from paralogues and ‘contaminating’ sequences based on analysis of Mendelian behaviour of DArTseq markers in thousands of control crosses in large diversity of organisms.

DNA concentrations were quantified using a Qubit 2.0 Fluorometer (Thermo Fisher Scientific) and adjusted to 50–100 ng µl^−1^ in a minimum volume of 10–20 µl—the optimal range for DArTseq. Samples less than 30 ng µl^−1^ were concentrated using Ambipure magnetic beads to avoid salt carryover. All samples included in assays had a final DNA concentration of greater than 30 ng µl^−1^.

### 2.3. DArTseq assays

DArTseq™ represents a combination of DArT complexity reduction methods and NGS platforms [9,14,15,20,21]. The technology is optimized for each organism and application in order to select the most appropriate complexity reduction method. Four combinations of enzymes (PstI/HpaII, PstI/SphI, SbfI/HpaII, SbfI/MseI) were tested in a pilot study to select the most appropriate complexity reduction method, both in terms of the size of the representation and the fraction of a genome selected for assays. Based on locus coverage, reproducibility and polymorphism (data not presented), the enzyme combinations of PstI/HpaII were selected for *C. caudicinctus* and PstI/SphI for *Litoria*.

DNA samples were processed in digestion/ligation reactions as described previously [20] except that the single PstI-compatible adaptor was replaced with two different adaptors corresponding to the PstI and SphI (or HpaII, in the case of *C. caudicinctus*) restriction enzyme overhangs. The PstI-compatible adapter was designed to include the Illumina flow cell attachment sequence, sequencing primer and a ‘staggered’, varying length barcode region, similar to the sequence previously reported [22]. The SphI-compatible adapter simply comprised the Illumina flow cell attachment region and SphI overhang sequence. Ligated fragments with both a PstI and SphI adapter were amplified by PCR using an initial denaturation step of 94°C for 1 min, followed by 30 cycles with the following temperature profile: denaturation at 94°C for 20 s, annealing at 58°C for 30 s and extension at 72°C for 45 s, with an additional final extension at 72°C for 7 min. Equimolar amounts of amplification products from each sample were combined before single end sequencing for 77 cycles on an Illumina Hiseq2500.

Sequences generated from each lane were processed using proprietary DArT analytical pipelines. In the primary pipeline, the fastq files were processed to filter out poor-quality sequences, applying more stringent selection criteria to the barcode region than the rest of the sequence. In that way, the assignments of the sequences to specific samples carried in the ‘barcode split’ step are very reliable. Approximately 2 500 000 (±7%) sequences per barcode/sample are used in marker calling in a high-density array, while a more cost-effective version of the assay using an average of 1.3 million/sample was also trialled. Finally, identical sequences were collapsed into ‘fastqcall files’. In summary, the primary pipeline filters poor-quality sequences while simultaneously applying more stringent selection criteria to the barcode region, ensuring the reliable assignment of sequences to specific samples, and then collapses identical sequences into ‘fastqcall’ files. These are used in the secondary pipeline for DArT P/L's proprietary SNP and SilicoDArT (presence/absence of restriction fragments in representation) calling algorithms (DArTsoft14). The data were converted to a matrix of SNP loci by individuals, with the contents stored as integers 0, homozygote, reference state; 1, heterozygote; and 2, homozygote for the alternate state.

We assess the quality and informativeness of the SNP datasets by means of reproducibility and polymorphism information content (PIC). The reproducibility score of markers is the proportion of technical replicate assay pairs for which the marker score is consistent. In diversity analysis, the reproducibility parameter threshold is set usually at 97% which translates to average reproducibility of the dataset around 99.7%. The PIC is an index for evaluating the informative extent of an SNP marker, with zero indicating no allelic variation and a maximum of 1.0 for absolute allele variation.

### 2.4. Data analyses

The proportion of missing data and heterozygosity per locus and per sample were also calculated to evaluate possible bias. We used Plink 1.9 (https://www.cog-genomics.org/plink2; [23]) and fastSTRUCTURE v. 1.0 [24], a Bayesian model-based clustering algorithm to infer population structure from large SNP genotype datasets. To facilitate data preparation and analysis, we designed a docker-based workflow termed ‘lizards-are-awesome’ (LAA). LAA minimizes the manual labour involved in preparing DArTseq SNP data in single row format for analysis with Plink and fastSTRUCTURE. Input data will be the metadata provided by DArTseq, saved as an xlsx file: ‘0’, reference allele homozygote; ‘1’, alternate allele homozygote; ‘2’, heterozygote and ‘-’, fragment missing in representation—double null (absence of fragment with SNP in genomic representation). LAA converts these data into ped and map files for Plink analysis. In addition to the conversion operation, LAA automatically initiates the program Plink on the generated ped and map files, and the resulting bed, bim and fam files are then passed on to and analysed with fastSTRUCTURE. The user can choose a maximum of *K* (number of populations) to be analysed by fastSTRUCTURE, as well as additional parameters. Output files include the mean *Q* value for each individual, defining the mean probability to belong to any one of the populations *K*1 to *Kx*. LAA packages and further details are available at https://github.com/furious-luke/lizards-are-awesome.

For each case study, analyses were run only for SNPs with a greater than 95% genotype call, i.e. the respective DNA fragment had been identified (=called) in greater than 95% of all individuals. Analyses in fastSTRUCTURE were repeated for a range of *K* (number of populations) for each study species: *C. caudicinctus—K*2–9; *Litoria* spp.*—K*1–5. We assessed the model complexity and model components for each analysis of *K* to determine if they both resolved the same most likely number of clusters (as recommended [24]). If not, the true *K* was determined as a value between the estimates predicted by fastSTRUCTURE and based on what made most biological sense.

GenAlEx 6.503 [25] was used to calculate frequency-based estimates (*F*-statistics, heterozygosity) and a distance-based principal coordinates analysis (PCoA), using the codominant genetic distance matrix generated from the SNPs, to elucidate the genetic relationships between the samples. A covariance-standardized PCoA was selected in GenAlEx 6.503, and a scree plot of resulting Eigenvalues was used to determine the number of PC axes to be used. Additionally, for the *C. caudicinctus* samples (*N* = 91), we explored phylogeographic relationships using a conditional likelihood approach [26], using SNPs on their own after excluding invariant sites. SNP data were converted to base pairs, then concatenated and stored in a PHYLIP format. Heterozygous SNPs were coded as the appropriate IUPAC ambiguity codes. A RaxML analysis was conducted on the CIPRES portal (https://www.phylo.org/), with the Lewis-type ascertainment bias correction selected. We used the K80 model of nucleotide substitution without rate heterogeneity, which was determined using jModelTest. This approach takes into account that no invariant sites are included in the data and helps reduce overestimation of tree lengths. Such approaches have been reviewed using simulations [26] and have found that although a ‘reconstituted DNA approach’ including invariant sites performed best, a conditional likelihood approach with an ascertainment bias correction (as we used) provide a viable alternative and allow phylogenetic analysis of exceptionally large datasets that are often prohibitively slow.

## 3. Results

### 3.1. Case study 1: *Ctenophorus caudicinctus*

A total of 28 960 SNPs were obtained using a DArTseq low-density assay, with an average genotype call rate of 75.4%, a scoring reproducibility of 99.7% and an average PIC of 0.19. A total of 309 SNPs had a 100% genotype call rate, while 1485 SNPs had a call rate of 95%. Assessment of analyses run on the three datasets (all SNPs, SNPs with 95% and SNPs with 100% call rates) indicated qualitatively similar results between the 95% dataset (1485 SNPs) and all SNPs (28 960 SNPs), while a loss of resolution was observed in the dataset based on 100% genotype call rates (309 SNPs). Here, we present the results from the 95% call rate dataset.

**Figure 3.**
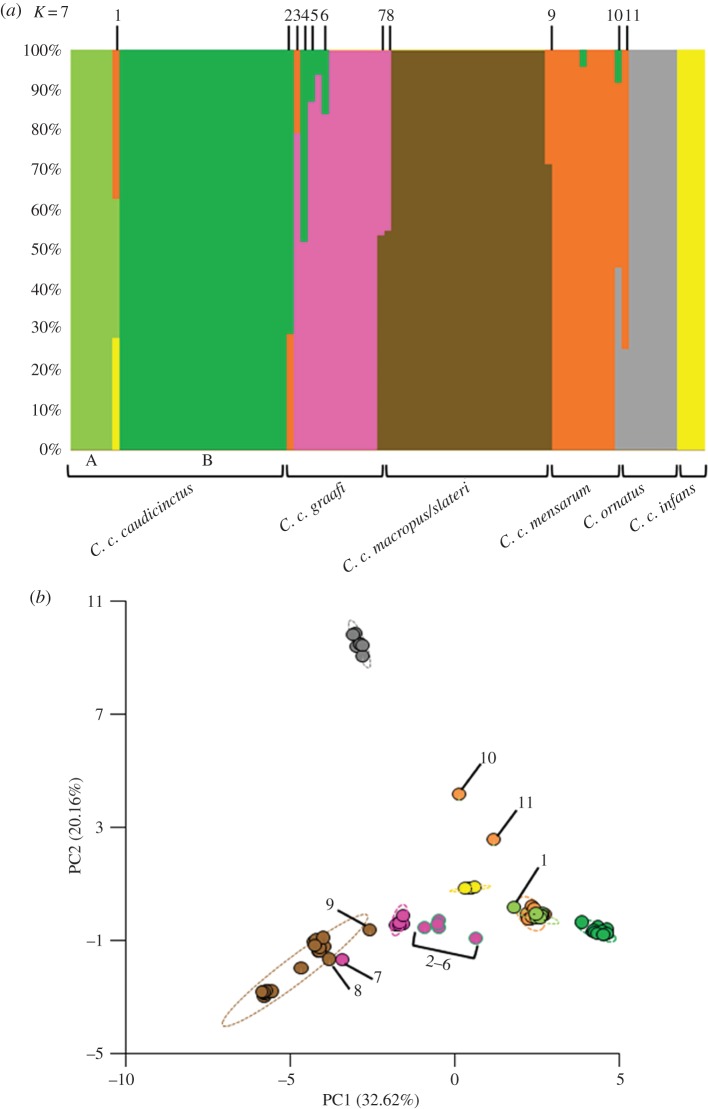
Population genetic analyses of 1485 SNPs for *C. ornatus* and the six subspecies of *C. caudicinctus*: (*a*) a fastStructure plot (with the simple prior) at *K* = 7; and (*b*) a distance-based (PCoA) plot of genetic structure, based on genetic distance between samples. Each of the *C. caudicinctus* subspecies and *C. ornatus* are coloured to match those in figure 1. Individuals identified as admixed in the fastSTRUCTURE analysis are indicated by a number 1–11 in both plots. Percentage of genetic distance explained by each of the PCoA axes (PC) are provided in parentheses.

A fastSTRUCTURE analysis resolved the most likely number of clusters at *K* = 7 (figure 3), which equates to samples being assigned to *C. ornatus*, to each of the five putative subspecies within *C. caudicinctus* (*C. c. caudicinctus*, *C. c. mensarum*, *C. c. infans*, *C. c. macropus/slateri* and *C. c. graafi*), and to *C. c. caudicinctus* which is further divided into two clusters. The smaller cluster within *C. c. caudicinctus* (Group A—figure 3) is geographically restricted to the central coastline of the Great Sandy Desert. We found evidence of hybrids between these seven clusters, with individuals classified as admixed between two or more clusters when 0.90 > *Q* ≥ 0.10 (*Q* = admixture proportion) for multiple clusters. Ten samples of the 91 were identified as being admixed (10.99%), with four individuals having between 45 and 55% contributions from each cluster, indicating F_1_ hybrids. Five individuals had *Q* values greater than 0.70 for one cluster and less than 0.30 for a second, indicative of backcrossing, while one individual had between 28 and 37% contribution from three clusters, indicating a possible hybridization between two subspecies followed by backcrossing with a third. Individuals that had been identified as showing introgression and/or gene exchange in previous research [16] were found to be probable backcrossed individuals, with the majority of the genetic signature coming from *C. c. graafi* (figure 3). One of these individuals (WAMR122612) had only a 6% genetic contribution from *C. c. caudicinctus* and as such was not included in the admixed individuals detailed above.

Average observed heterozygosity for genetic clusters (*K* = 7) was low, ranging from 0.011 to 0.015, but was not consistently higher or lower for a given cluster. Differentiation between the clusters was found to be significant (*F*_st_ = 0.247, *p* = 0.001), indicative of deep phylogeographic structure. An AMOVA showed that 25% of the total molecular variance was due to differences between the clusters, whereas 62% were partitioned between individuals. A PCoA exhibited strong differentiation between the seven genetic clusters, excluding admixed individuals, on PC1 (*F*_6,79_ = 533.5, *p* < 0.0001; figure 3) and PC2 (*F*_6,79_ = 247.0, *p* < 0.0001; figure 3). The first two principal coordinates of PCoA accounted for 33% and 20% of the variance, respectively, thus jointly accounting for 53% of the total variation in the dataset. *Ctenophorus ornatus* was significantly differentiated from all of the *C. caudicinctus* genetic clusters on PC2 (Tukey's post hoc test; *p* < 0.0001) but not on PC1. Variance of PC1 and PC2 scores, excluding admixed individuals was greater in *C. c. macropus/slateri* (PC1 = 0.911; PC2 = 0.728) than all other genetic clusters.

**Figure 4.**
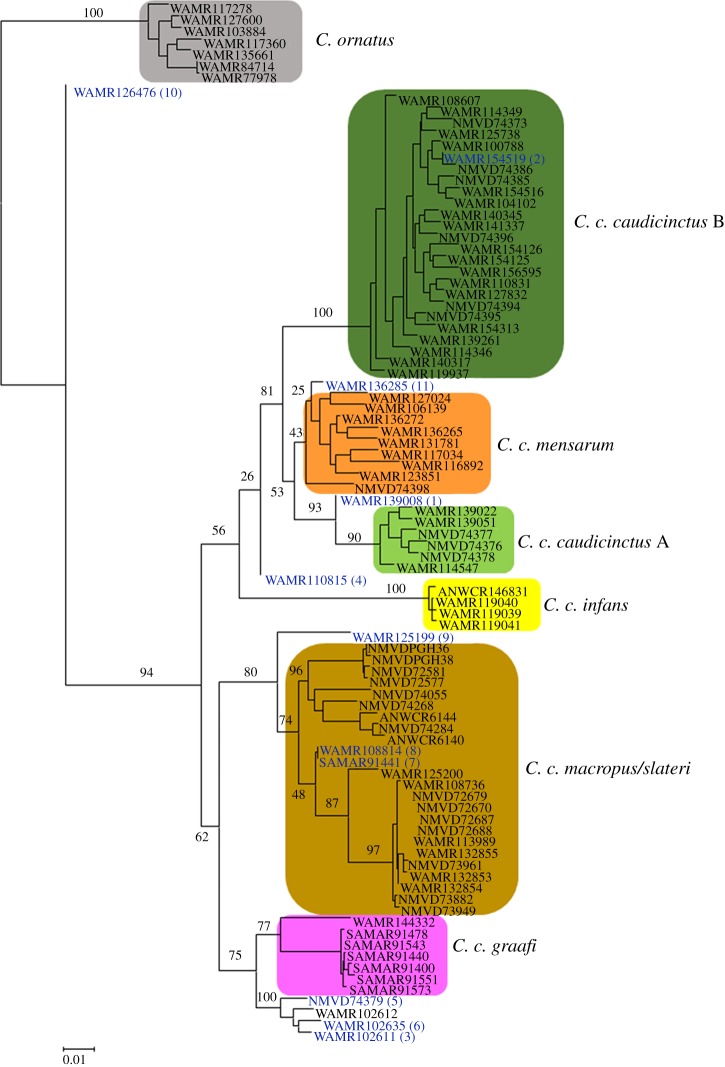
Conditional maximum-likelihood phylogenetic tree for *C. ornatus* and the six subspecies of *C. caudicinctus* based on 1485 SNPs. ML bootstraps are provided about the branches. Colours designate clades identified in the fastStructure analysis (figure 3). Admixed individuals are coloured blue and numbered to correspond to those identified in figure 1.

We also explored phylogenetic relationships between the 91 individuals using a conditional likelihood approach (figure 4). The same major clusters were evident as in both the fastSTRUCTURE and PCoA analysis, with *C. ornatus* highly supported (bootstrap 94%) as the sister species to all the *C. caudicinctus* subspecies. Within *C. caudicinctus*, there is an eastern clade (*C. c. macropus/slateri* and *C. c. graafi*) and a western clade (*C. c. caudicinctus* A, *C. c. caudicinctus* B, *C. c. mensarum, C. c. infans*), although the monophyly of these two geographical lineages is not strongly supported. The individuals identified as being admixed in the fastSTRUCTURE analysis occur throughout the phylogeny and, in most cases, fall outside the subspecies clades. The exceptions to this are admixed individuals #2, #7 and #8 (figure 4). Phylogenetic relationships are similar to those identified in the previously published phylogeography [16]; however, mtDNA (figure 1) resolved *C. ornatus* as being nested within *C. caudicinctus* and aligned to the western lineage. Additionally, the phylogeny based on SNPs provides far greater resolution than that based on five nuclear genes [16].

### 3.2. Case study 2: *Litoria ewingii*–*Litoria paraewingi*

A total of 48 117 SNPs were obtained using a DArTseq low-density assay, with an average genotype call rate of 79.3%, a scoring reproducibility of 99.9% and an average PIC of 0.18. A total of 1307 SNPs had a 100% genotype call rate, while 5278 SNPs had a call rate of 95%. In a high-density assay, a total of 67 060 SNPs were obtained, with an average genotype call rate of 78.8%, a scoring reproducibility of 99.9% and an average PIC of 0.18. A total of 1663 SNPs had a 100% genotype call rate, while 6732 SNPs had a call rate of 95%. Assessment of analyses run on the low- and high-density arrays indicated qualitatively similar results; thus, we present the results from the 95% call rate, low-density array dataset. Results from the high-density array are provided in electronic supplementary material, figure S1.

**Figure 5.**
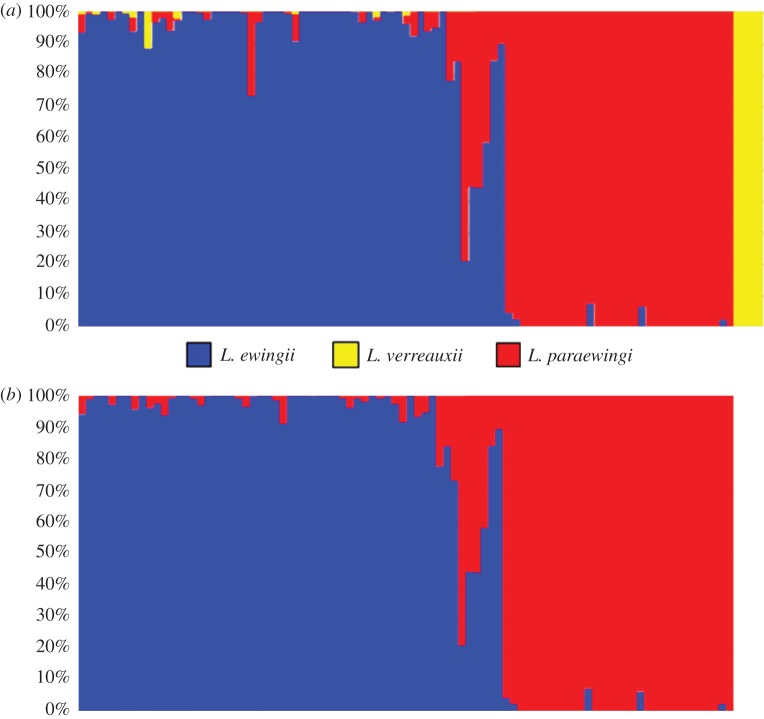
Population genetic analyses of 5278 SNPs resulting from a low-density array for *L. ewingii* and *L. paraewingi*: (*a*) a fastStructure plot (with the simple prior) of samples including *L. verreauxii* at *K* = 3; and (*b*) a fastStructure plot (with the simple prior) of samples excluding *L. verreauxii* at *K* = 2.

Average observed heterozygosity ranged from 0.11 to 0.16 but was not consistently higher or lower for a given species. An initial fastSTRUCTURE analysis of all 94 samples resolved the most likely number of clusters at *K* = 3 (figure 5), which equates to samples being assigned to each of the three species, *L. ewingii*, *L. paraewingi* and *L. verreauxii*. In this analysis, four samples were identified as pure *L. verreauxii* (*Q* > 99%), and one sample was identified as a hybrid between *L. verreauxii* (*Q* = 12.5%) and *L. ewingii* (*Q* = 87.5%). These five samples were removed from the dataset and a subsequent fastSTRUCTURE analysis identified *K* = 2 as the most likely number of clusters (figure 5). There was evidence of hybridization between the two species, *L. ewingii* and *L. paraewingi*, with individuals classified as admixed between the species when 0.92 > *Q* > 0.08, as described in the previous *Litoria* spp. microsatellite study [17]. Eleven samples of the 88 were identified as admixed (12.5%), with three individuals having between 44 and 56% contributions from each species, indicating possible F_1_ hybrids. All eight remaining hybrids had *Q* values greater than 0.70 for one species, indicative of backcrossing. Only five of these 11 hybrid individuals had been identified as admixed in previous research using microsatellite markers, with the other six classified as ‘pure’ in the microsatellite study [17]. Furthermore, 12 individuals identified as hybrids using microsatellites were not designated as admixed in our fastSTRUCTURE analysis. Thus, the percentage of admixed individuals identified in the DArTseq low-density assay is lower (12.5%) than that based on previous microsatellite results (19.32%).

A comparison of fastSTRUCTURE results based on the low (figure 5) and high (electronic supplementary material, figure S1) density arrays shows that all individuals identified as hybrids in the low-density array were equally identified in the high-density array. One additional individual was identified as a hybrid in the high-density array (*Q_K_*_1_ = 0.08, *Q_K_*_2_ = 0.92), while in the low-density array, this individual was assigned to population *K*2 (*Q_K_*_1_ = 0.06, *Q_K_*_2_ = 0.94).

**Figure 6.**
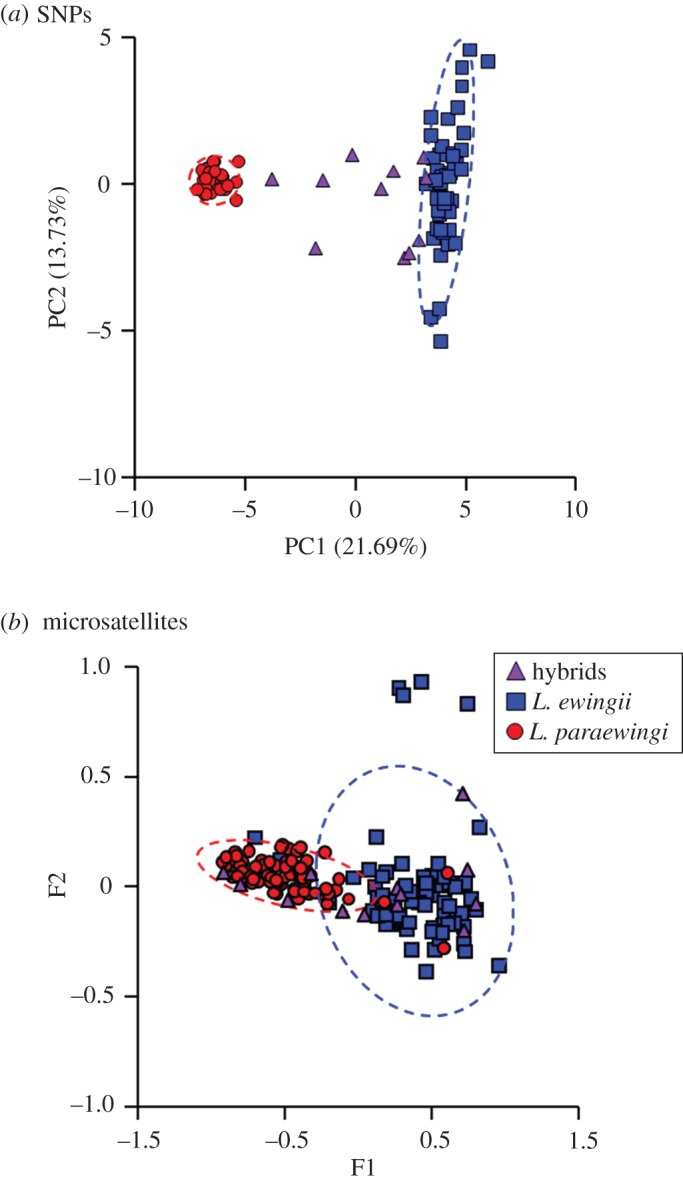
Multi-variate plots of genetic structure *L. ewingii* and *L. paraewingi*: (*a*) PCoA analysis based on genetic distance between samples of 5278 SNPs, resulting from a low-density array; and (*b*) a plot of a previously published multiple correspondence analysis (MCA) based on microsatellite data [17]. Dashed lines indicate 90% confidence ellipses for each parental species. Purple triangles indicate hybrid individuals. Percentage of genetic distance explained by each of the PCoA axes (PC) are provided in parentheses.

Between the two species (*L. ewingii*, *L. paraewingi*) and the hybrid individuals identified in the fastSTRUCTURE analysis, average heterozygosity was comparable (0.15, 0.11, 0.16, respectively). Differentiation between the groups was not found to be significant (*F*_st_ = 0.001, *p* = 0.34), indicative of hybridization. An AMOVA showed that none (0%) of the total molecular variance was due to differences between the three groups, whereas 36% were partitioned between individuals. A PCoA exhibited strong differentiation between the parental species (*F*_1,75_ = 7806.8, *p* < 0.0001; figure 6) on PC1, as opposed to PC2. The first two principal coordinates of PCoA accounted for 22% and 14% of the variance, respectively, thus jointly accounting for 36% of the total variation in the dataset. Variance of the PC2 scores for *L. ewingii* (4.35) was higher than for *L. paraewingi* (0.12). In good agreement with the previous study based on microsatellites [17], the PCoA of SNPs found that the majority of admixed genotypes (eight out of 11) were more similar to *L. ewingii* than to *L. paraewingi*, implying that backcrossing most often involves *L. ewingii* and/or that *L. ewingii* backcrosses have higher fitness.

## 4. Discussion

Our study highlights the power of the DArTseq platform to provide insights into the genetic structure of vertebrate systems at varying evolutionary and geographic scales. Genome complexity reduction approaches, including DArTseq, provide advantages over whole-genome sequencing such as the increased depth of coverage per locus, which improves the rate of genotype calling and the ability to sequence more samples for less cost [6]. In addition, such platforms can be applied to systems where no reference genomes are available. For these reasons, double-digest restriction fragment sequencing is particularly appropriate to evolutionary genetic studies in natural systems. In our study, the DArTseq platform provided thousands of markers (5278 SNPs with 95% coverage) across the genome without the requirement of a sequenced reference genome in the case of the *Litoria* system. At the same time, it provided the opportunity to map the markers identified for *C. caudicinctus* back to an assigned reference genome, in this case, the recently published genome of the agamid lizard *Pogona vitticeps* [27].

### 4.1. Identification of hybridization and admixture

Genome reduction approaches, particularly ddRAD, are being increasingly used in studies of hybridization and admixture in vertebrates [28–30]. To date, the DArTseq platform has infrequently been used in vertebrate systems. Recent studies successfully applied DArTseq to resolve phylogenetic relationships in a ray [11], develop SNPs for paternity testing in mosquitofish [12] and population genetics in fish [14,15]. We identified admixture using fastSTRUCTURE [24], which provides variational algorithms that are almost two orders of magnitude faster than STRUCTURE [31] and achieve accuracies comparable to those of ADMIXTURE [32]. Our study demonstrates its utility in both a classic hybrid system and a phylogeographic context.

We were able to identify admixture and hybrids with greater resolution than was achieved using traditional methods. The previous phylogeographic study of *C. caudicinctus* [16] proposed the presence of admixture between deeply divergent mtDNA-based clades, but the lack of resolution in five nuclear genes meant that this could not be confidently confirmed. We used the DArTseq platform to further investigate this question and were able to confirm admixture between clades. Remarkably, admixture was detected even between *C. caudicincuts* and *C. ornatus* (figure 3), for which we had previously described greater than 13% mtDNA pairwise divergence [16]. A comparison of the different analytical approaches we used for the SNP data demonstrates the importance of using population genetic analyses (fastSTRUCTURE and PCoA) in addition to phylogenetic approaches (conditional maximum likelihood) when admixture may be present, as the phylogenetic approach (figure 4) failed to distinguish all admixed *Ctenophorus* individuals.

Similarly, in case study 2 on the well-studied hybrid zone between the two *Litoria* frog species, we found that the DArTseq platform significantly increased the resolution in identifying hybrids and admixture between species when compared with previous microsatellite data. There was a clear definition between the parental species, and most hybrids fell outside the 90% confidence ellipses (figure 6), with only a couple of probable backcrossed individuals falling within the 90% confidence ellipse. To gain further insight into the hybridization of these frogs, such as identifying F_1_ versus backcrossed individuals, other analytical approaches need to be taken. NewHybrids is able to identify F_1_ versus backcrossed progeny; however, it is currently unable to deal with a dataset with 1000s of SNPs. In a recent study, the number of SNPs analysed was reduced to 200, from a dataset of greater than 4000 SNPs to allow researchers to implement NewHybrids [11]. Despite these analytical issues, the greater resolution provided by the DArTseq platform will allow analysis of this study system that has not been possible with microsatellites. In particular, mapping of phenotypic traits, such as variation in call structure across the hybrid zone. We will also be able to further explore, at a genomic level, the one-way genetic incompatibility between *L. ewingii* and *L. paraewingi*, which leads to 67–100% of progeny developing anophthalmia, a lethal developmental condition, in a female *L. paraewingi* and male *L. ewingii* cross [17]. Until now, such questions could not be explored using the existing microsatellite data in this study system.

Although further investigating the genomic architecture of gene exchange in hybrid zones and mapping phenotypic traits across these areas of admixture is very appealing, recent work has highlighted that caution is required with using genome reduction systems when looking for loci under selection. A recent study concluded that genome scans based on RADseq data alone, while useful for studies of neutral genetic variation and genetic population structure, are likely to miss many loci under selection in studies of local adaptation [33]. In addition, it has been suggested that if genome-wide linkage disequilibrium is low, as is the case in many species with large population sizes, most genome subsampling methods will not sample densely enough to detect selected variants [34]. These researchers suggest that whole-genome resequencing methods, instead, will allow phylogeographers to identify loci involved in phenotypic divergence and speciation. The DArTseq platform has been used to develop dense genetic linkage maps and for mapping QTL for traits in plants [35,36], but further investigation in the utility of low- versus high-density DArTseq arrays in studies of loci associated with phenotypic variation in vertebrate systems is still required.

### 4.2. Sample preparation and quality

High-quality and an appropriate quantity of genomic DNA is crucial to the success of these genome-wide approaches [7]. However, when assessing natural populations, researchers are often forced to work in conditions that are not ideal, collecting suboptimal tissue types and quantities [37]. Sample contamination can pose a particular problem to NGS approaches, and is particularly significant in aquatic and marine organisms [18,19], where samples collected in wild populations are exposed to water contaminated with microbial DNA. In our second case study, toe-clips from frogs posed such a problem, with cleaning of the surfaces prior to sample collection not being wholly successful. Consequently, we employed downstream protocols to screen out microbial genetic contamination. The DArTsoft14 software distinguishes allelic sequence variants from paralogues and ‘contaminating’ sequences based on analysis of Mendelian behaviour of DArTseq markers in thousands of control crosses in large diversity of organisms, thus, ‘filtering’ of viral and/or bacterial sequences in SNP marker selection. As detailed in our methods, subsequent BLAST search of marker sequences against bacterial and viral databases yielded no hits, indicating successful filtering of microbial contaminants. Mendelian inheritance filters have successfully been used to improve SNP discovery in both model and non-model species using GBS platforms [38]. Such an approach provides greater confidence that data collected in natural settings, where environmental contamination is a problem, can still be used with an NGS approach.

An additional problem for many NGS approaches is the quantity of sample required [7]. Most population genetic studies on vertebrates now use non-invasive or at least non-lethal tissue-sampling methods, which may substantially reduce DNA quantity and possibly quality. This is particularly relevant on studies of threatened or vulnerable species. Unlike some NGS approaches, genome complexity reduction approaches, such as DArTseq, can be performed even with limited amounts of genomic material, allowing application to a great range of sample types. For DArTseq, it is recommended that samples contain 10–20 µl of an aqueous solution of high-quality DNA at 50–100 ng µl^−1^. All samples from our study passed quality checks and we had a very low proportion of samples that failed to be genotyped (97% success *C. caudicinctus*; 100% success *Litoria* spp.). Some of the samples from case study 2 (*Litoria* spp*.*) were at lower concentrations than recommended, although we ensured all samples contained at least 30 ng µl^−1^. Despite the lower than recommended DNA concentrations in this case study, all samples were successfully genotyped. Using the toe-clips from small frogs did make achieving the optimal DNA concentrations a challenge, but we found that the extraction method made a significant difference to the quality and amount of DNA. We used samples that had been extracted via two standard extraction methods (chloroform : isoamyl alcohol procedure and commercially available kits) and found that for the frog toe-clips, those samples extracted using the chloroform : isoamyl alcohol procedure resulted in higher DNA yields than those extracted using kits (results not shown). We also concentrated samples using Ambipure magnetic beads to avoid salt carryover, which improved quality of the DNA for inclusion in DArTseq assays. Consequently, we recommend selecting extraction methods that optimize DNA yield and quality when using small, finite tissue samples, such as toe-clips.

## 5. Conclusion

Although DArTseq has been used extensively in commercially important plant species, its use in natural systems in vertebrates has been limited and has been mostly focused on population genetics and phylogenetics [11,13–15]. However, until now the utility of DArTseq in identifying hybridization and admixture in natural systems has not been assessed. We show that it provides a promising approach for vertebrates, in addition to other genome reduction approaches. Population genetics and phylogeographic research have shown that many species consist of multiple, highly divergent genetic lineages, with evidence of hybridization and introgression between these lineages. Using this NGS approach, we increased resolution in the identification of hybrids and admixed individuals when compared with traditional molecular approaches, and also provided insight into past gene flow and introgression between populations at a phylogeographic level. We conclude that DArTseq is a platform that will be of particular interest to researchers working at the interface between population genetics and phylogenetics, exploring species boundaries, gene exchange and hybridization.

## Supplementary Material

Population genetic analyses of high density array

## Data accessibility

Additional analyses can be found in the electronic supplementary material. The SNP data files are available in the Figshare digital repository and can be accessed at: Litoria_95%_lowdensity (https://figshare.com/s/7655985cd9608d80009a); Litoria_95%_highdensity (https://figshare.com/s/d53f55d494dfc2632928); Caudicinctus_95%_lowdensity (https://figshare.com/s/e1678763ecfbf1a41cb6).
