# Supplementary material for: Identifying hybridization and admixture using SNPs: application of the DArTseq platform in phylogeographic research on vertebrates
Source: R Soc Open Sci. 2017 Jul 19;4(7):161061. doi: 10.1098/rsos.161061 (PMC5541528; doi:10.1098/rsos.161061)
Supplement: Population genetic analyses of high density array [file rsos161061supp1.pdf]

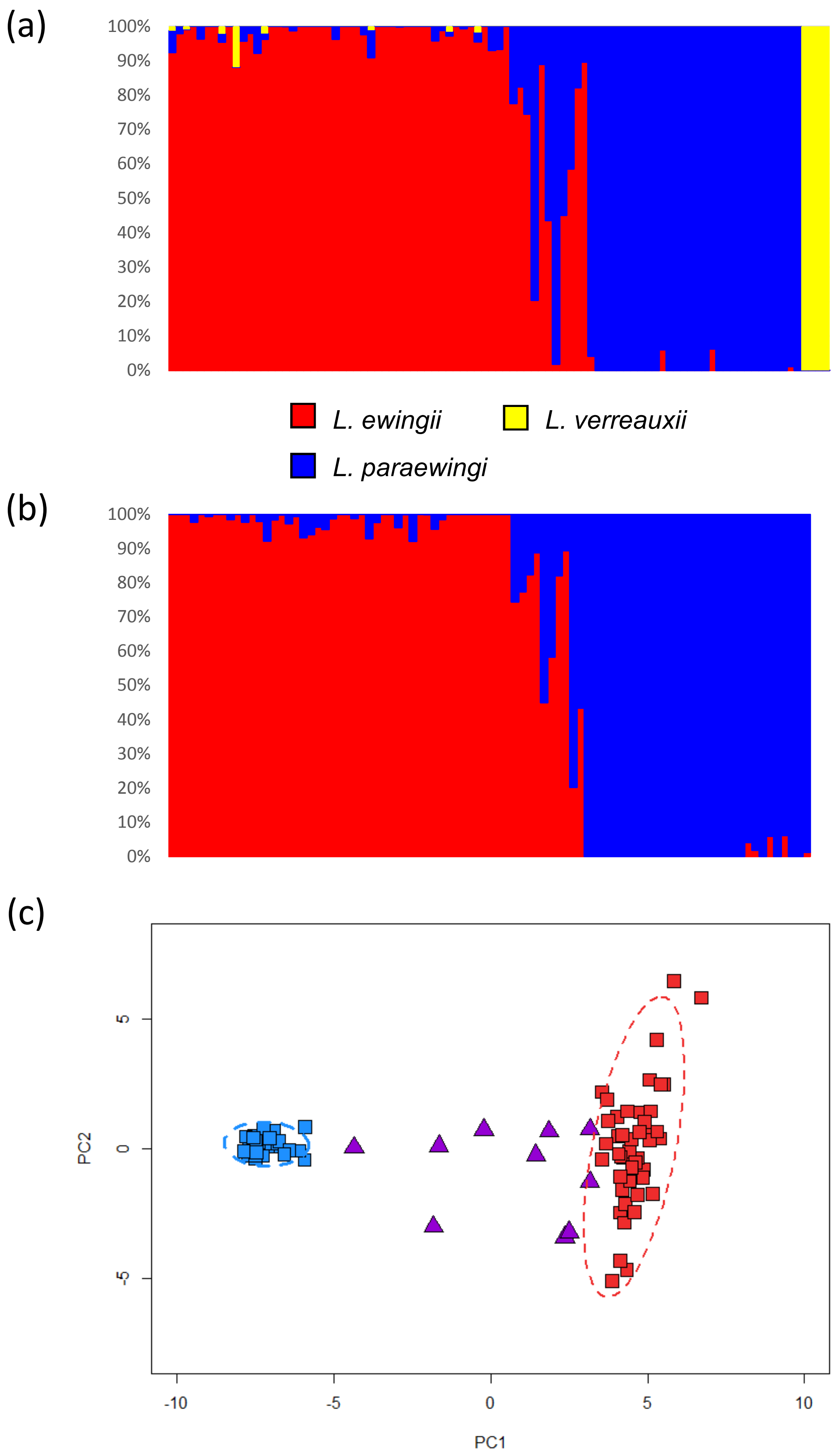

Fig. S1 Population genetic analyses of 6738 SNPs resulting from a high density array for *Litoria ewingii* and *L. paraewingi*: (a) a fastStructure plot (with the simple prior) of samples including *L. verreauxii* at K = 3; (b) a fastStructure plot (with the simple prior) of samples excluding *L. verreauxii* at K = 2; and (c) PCoA analysis, with 95% confidence ellipses shown and hybrid individuals represented as purple triangles.
